# The Challenge by Multiple Environmental and Biological Factors Induce Inflammation in Aging: Their Role in the Promotion of Chronic Disease

**DOI:** 10.3389/fimmu.2020.570083

**Published:** 2020-10-14

**Authors:** María Consuelo Bachmann, Sofía Bellalta, Roque Basoalto, Fernán Gómez-Valenzuela, Yorschua Jalil, Macarena Lépez, Anibal Matamoros, Rommy von Bernhardi

**Affiliations:** ^1^ School of Medicine, Pontificia Universidad Católica de Chile, Santiago, Chile; ^2^ Institute of Biological Sciences (ICB), Federal University of Pará, Belem, Brazil

**Keywords:** pollution, oxidative stress, nutrition, immune system, gender, exercise, epigenetic changes, drug abuse

## Abstract

The aging process is driven by multiple mechanisms that lead to changes in energy production, oxidative stress, homeostatic dysregulation and eventually to loss of functionality and increased disease susceptibility. Most aged individuals develop chronic low-grade inflammation, which is an important risk factor for morbidity, physical and cognitive impairment, frailty, and death. At any age, chronic inflammatory diseases are major causes of morbimortality, affecting up to 5–8% of the population of industrialized countries. Several environmental factors can play an important role for modifying the inflammatory state. Genetics accounts for only a small fraction of chronic-inflammatory diseases, whereas environmental factors appear to participate, either with a causative or a promotional role in 50% to 75% of patients. Several of those changes depend on epigenetic changes that will further modify the individual response to additional stimuli. The interaction between inflammation and the environment offers important insights on aging and health. These conditions, often depending on the individual’s sex, appear to lead to decreased longevity and physical and cognitive decline. In addition to biological factors, the environment is also involved in the generation of psychological and social context leading to stress. Poor psychological environments and other sources of stress also result in increased inflammation. However, the mechanisms underlying the role of environmental and psychosocial factors and nutrition on the regulation of inflammation, and how the response elicited for those factors interact among them, are poorly understood. Whereas certain deleterious environmental factors result in the generation of oxidative stress driven by an increased production of reactive oxygen and nitrogen species, endoplasmic reticulum stress, and inflammation, other factors, including nutrition (polyunsaturated fatty acids) and behavioral factors (exercise) confer protection against inflammation, oxidative and endoplasmic reticulum stress, and thus ameliorate their deleterious effect. Here, we discuss processes and mechanisms of inflammation associated with environmental factors and behavior, their links to sex and gender, and their overall impact on aging.

## General View

The systemic chronic low-grade inflammation observed in aged individuals has been coined as “inflammaging” (1), and leads to metabolic dysfunction, physical limitations, and frailty in older adults [reviewed in (2)]. Inflammaging leads to an increased secretion of interleukin 1beta (IL1β), interferons (IFNs), and tumor necrosis factor α (TNFα) (3). This inflammatory response appears to depend on biological factors like sex, being higher in older women, and is influenced by many environmental factors. The environment affects multiple biological mechanisms, epigenetics, mitochondrial function, cellular senescence, proteostasis, intercellular communication, metabolism, and inflammation (4). Several environmental links to chronic inflammation and age-related diseases have been shown including cardiovascular disease, type-2 diabetes, hypertension, and neurodegenerative disease (5). Their influence on chronic inflammation impacts aging across the individual’s lifespan [reviewed in (6)] and can have both beneficial and deleterious effects. For example, nutritional factors like western diet, associate with DNA damage and the impairment of its repair [discussed in (7)]. By the contrary, the Mediterranean diet shows beneficial effects, such as reduced inflammation, cardiovascular disease, and mortality (8). Several cytokines linked to inflammation, some of which target the nuclear factor kappa-light-chain-enhancer of activated B cells (NFκB) pathway [reviewed in (9)] are also associated with metabolic changes [discussed in (10)]. Metabolic interventions like caloric restriction extends health- and lifespan in *C. elegans*, *Drosophila*, and mice.

Aging has also been linked to epigenetic changes affecting the regulation of expression of many genes. Several of the epigenetic mechanisms, including DNA methylation, histone modifications and miRNAs, are sensitive to the environment, change with age (11) and depend on the sex of the individual. Gene-environment interactions have been found in genes coding for proinflammatory cytokines such as IL1β and air pollution, being correlated to inflammation and increased risk of Parkinson’s disease (12).

Human aging and age-related chronic diseases have been linked to mitochondrial impairment (13), with decreased energy production and increased generation of radical oxygen species (ROS), and inflammation (14). Because inflammation appears to be involved in the molecular, phenotypic, and functional consequences of aging, a potential strategy to tackle pathological aging could be to intervene the inflammatory state of aging. Reducing chronic inflammation could prevent pathological aging phenotypes and their functional consequences. One readily accessible place for intervention is the modification of deleterious lifestyle factors. Obesity, for example, has a strong correlation with systemic inflammation, and together with insulin resistance are frequently observed in aging. Unhealthy diet, stress, use of drugs, exposure to pollution and sedentarism can lead to obesity, defective immunoregulation, and inflammatory cytokines production (15–17). Pollutants, especially those that are stored in adipose tissue, affect both inflammatory and metabolic pathway genes (18). In addition to health behavior (sleep, diet, physical activity), and exposure to environmental toxins [reviewed in (19)], an important, although lesser studied, part of the environmental context are social interactions. Exposure to psychosocial stress (20) and poor sleep (21) can contribute to elevated inflammation. Many sources of stress, not just traumatic events, may have pathophysiological implications, contributing to dysregulate the immune response, and have long lasting effects on aging.

The impact of environmental factors will depend on the combination of specific conditions, their timing along the lifespan, and their interaction with biological factors such as sex and the individual genetic background. Thus, aging and the risk for chronic diseases is the result of the combination of multiple factors. Understanding the interplay of these factors may offer the opportunity to design specific interventions.

## Sex-Dependent Changes on Immune-Inflammatory Response

The inflammatory response is different in men and women. Adult females develop stronger innate and adaptive immune responses than males. These sex-related differences can determine the ability of immune cells to generate an effective inflammatory response, which translates into epidemiological differences on the prevalence of various pathologies, including allergies (22), asthma (23, 24), autoimmune diseases (25), anaphylaxis (26), neonatal sepsis (27), and cancer (28), among several pathologies. The immune response of women is polarized towards an increased production of Th2 cells, T regulatory cells (Treg), M2 macrophages, IL4, IL10, and GATA-3 cytokines, and decreased Th1, Th17, TBet, and RORγt lymphocytes (29–31). On the contrary, men show an immune response that depends on Th1 lymphocytes (32, 33), high IL33 production (34) and low levels of reactive mast cells (35). Men have also an increased response of microglia in the central nervous system (CNS) and an increased presence of TNFα and prostaglandins in response to inflammatory stimuli (36).

### Mechanisms Involved in the Sex-Dependent Differences in Inflammatory Response

Differences in inflammatory response between men and women vary among specific tissues. In the CNS inflammation, women show greater levels of B-cell (CD19+, CD5+, CD1d^hi^ B10) migration from the spleen to the site of injury than men, followed by an increase of macrophages/microglia (CD11b+, CD206), which appears to generate a lower neuroinflammatory response in female compared with male mice (37). In addition, women develop an increased immunoreactivity due to high numbers of IFN-producing dendritic cells (38, 39). Female mice tend to have M2 phenotype and activated eosinophils and mast cells show a higher reactivity than in male mice (35, 40). However, in response to an acute inflammatory stimulus, males produce higher amounts of inflammatory cytokines, CD8a+ neutrophil and T cells infiltration of the injury site (41). Conversely, the inflammatory microenvironment in female mice is characterized by an increased production of antibodies (42, 43) and a differential pattern migration of antibody-secreting cells (42).

The immune system responds differently in men and women not only because of the influence of sex hormones, but also differences in the patterns of autosomal methylation and X chromosome methylation, which determine distinctive profiles of gene expression (43, 44). Sex hormones exert antagonist effects on the immune system: Both estradiol and testosterone have a suppressive effect on the immune response (45).

Estrogen is the sex hormone with the greatest impact on the immune response, being described as one of the non-modifiable regulators of the immune system, due to its immunoregulatory and protective effects in many inflammatory models (46). However, this is contradictory with the fact that women have a higher prevalence of autoimmune diseases than men, although estrogens should be a protective condition (47).

The sex-dependent difference in the immune response is time-, and estrogen dose-dependent (29). Variations on the estrogen concentration during the ovulatory cycle, puberty or menopause, can promote the development of immune-related diseases (48). Mice exposed to chronic estrogen-treatment generate hormone resistance, decreasing the clonal expansion of Treg lymphocytes in autoimmune diseases (49, 50).

Estrogen regulates immune response primarily through α- and β-estrogen receptors (ERα/β), mitogen-activated protein kinase (MAPK) pathways, estrogen-dependent 3′-5′-cyclic adenosine monophosphate (cAMP) response element-binding (CREB), and modifications in the production of cAMP in immune cells (51). In addition to estrogen receptors, the presence of IL receptors influences the type of immune response; female macrophages express greater amounts of IL4 receptors than males. IL4 receptors favor the M2 phenotype when stimulated by estrogen. In agreement with that, estrogen induces an increased expression of IL4 on naive CD4+ T cells (40, 52). For a better general view of estrogen´s mechanisms and effect on the innate immune system cells we recommend reviews that have extensively covered those topics (53–56).

### Dependence on the Sex of the Immune Response Gene Expression

Sex regulates gene expression in multiple human tissues, in fact, one third of the autosomal genes that are expressed in a sex-biased manner exhibit androgen or estrogen hormonal response elements (57, 58). Sex hormones play a strong role in sexually dimorphic gene networks (59), inducing aberrant expression in immune response genes *via* differential methylation CCL18 CXCL5 IL5 (60). There are changes in the methylation pattern of sex-dependent immune response genes during embryonic development, which are reinforced in puberty by the estrogen-mediated induction of active forms of chromatins that are maintained during adulthood (61).

Immune response-related genes located in chromosomes 3 and X are differently expressed in B lymphocytes depending on the sex of the individual (62). Among the differentially expressed genes that are relevant for the immune/inflammatory response, can be mentioned the Toll-like signaling, cytokine receptors, Jak-STAT pathway and genes related to the activation of T-cell receptors (63). Phenotypically, the different pattern of gene expression may explain the greater female T-cell expandable capacity when exposed to an antigen (64).

Female T cells present higher activation and division capacities than their male counterparts. However, male T cells can develop greater infiltration potential and a lower self-reactive phenotype than female ones (65, 66). These differences could be due to the high expression of peroxisome proliferator-activated receptors (PPARs) (64), prostaglandins, and cyclooxygenase-2 (COX-2) in males (67).

The influence of sex on the immune response is observed throughout life and is accentuated with aging. In the neonatal stage, women have a lower concentration of regulatory T lymphocytes than men (68). During childhood, men develop a more intense immune response and are more likely to develop infections by various pathogens compared with women (69, 70). With increasing age, the dynamics and proportion of lymphocytes and myeloid cells differ depending on the sex due to the differential expression of 144 genes of the immune response in men and women (71). Also, in aged individuals, epigenomic changes generate a more robust innate and pro-inflammatory response in men and an increased activity in the adaptive immune response in women (72, 73). In recent times, during the COVID-19 pandemic, it has been observed that the infection by SARS-CoV-2 in older adults shows conspicuous differences; men have elevated plasma levels of IL8 and IL18 and a high amount of monocytes whereas women develop a robust activation of T lymphocytes (74). This differences in the immune response could explain the higher mortality of COVID19 in men than in women (75, 76).

To recapitulate, sex hormones and genetic expression patterns in men and women can generate distinct immune and inflammatory responses that determine singularities in the epidemiological distribution of immune diseases. Research protocols in immune response and inflammation must be redefined to avoid results biased by sex. Furthermore, research in women is urgently needed to define the efficacy for women of several therapies that were originally tested in men.

## Nutrients and Inflammation: Role of the Diet and Polyunsaturated Fatty Acids

The increase in noncommunicable diseases (NCDs), such as obesity, hypertension and cancer as well as the low-grade chronic inflammation that characterizes most NCDs (77) can be affected by environmental factors that change the immune response. Lifestyle factors like nutrition can modulate the immune system. It has been reported in mice that western diet-induced systemic inflammation and reprogramming of myeloid cell precursors is mediated by the activation of the NLRP3 inflammasome, which is a key sensor of the innate immune system for metabolic danger signals, such as uric acid and cholesterol (78). Metabolic regulation appears to be very robust and long lasting, being reported that proper nutrition during pregnancy can reduce the risk for NCDs in the offspring even at adult age (79, 80).

### The Impact of the Diet on the Immune Response and Inflammation

Some diet types can result in metabolic and epigenetic changes that affect immune function (81), as reported in populations that consume a high-fat and low-fiber western diet, who show a prevalence of NCDs higher than populations that consume a Mediterranean diet or a diet based on bioactive compounds, like the hydroxytyrosol in olive oil (82–84). There is evidence supporting the anti-inflammatory activity of phenolic extracts from olive oil, such as their ability to reduce lipopolysaccharide (LPS)-stimulated Nitric oxide (NO) production by the RAW-264.7 macrophage cell line. The hydroxytyrosol stearate and the hydroxytyrosol oleate decrease NO production in a concentration-dependent manner (85). In addition, olive oil extracts increase total plasma glutathione concentration (86), increasing the antioxidative response of the individual.

Nordic diet has many similarities with the Mediterranean diet, but its effects on low-grade chronic inflammation are less known. Both diets include abundant fruits, vegetables, whole grain products, fish and vegetable oil, but restrict saturated fat and red and processed meats (87, 88). Observational (89, 90) and interventional (91, 92) studies report an inverse association between the adherence to Nordic diet and the concentration of high sensitivity C-reactive protein (hsCRP). Single intervention studies reported beneficial effects, reducing IL1 receptor α (IL1Rα) (87) and Cathepsin S (93), and downregulation of inflammatory mediators in the adipose tissue (94) and peripheral blood mononuclear cells (PBMCs) (95). A key nutrient in fish are the n3 polyunsaturated fatty acids (PUFAs) (88). The Greenland Inuit population, which has a high dietary intake of n3-PUFAs, have a lower incidence of myocardial infarction than the Danish population (96). Numerous studies associate the cardioprotective effects of n-3 PUFAs to their effect on immunomodulation (97–99), and control of inflammation, including neuroinflammation during aging (100).

### The Mechanism of the Anti-Inflammatory Effects of n3-PUFAs

n3-PUFAs can regulate the transcription and expression of inflammatory mediators such as cytokines, chemokines and adhesion molecules in cardiomyocytes, fibroblasts, endothelial cells, and monocyte-macrophages (101–104). Anti-inflammatory effect of eicosapentaenoic acid (EPA), docosahexaenoic acid (DHA) and their biologically active metabolites (D and E Resolvins - mediators derived from omega-3 fatty acids, primarily EPA and DHA that block the production of proinflammatory mediators and regulate leukocyte trafficking to inflammatory sites) can be mediated through one of the mechanisms capable of reducing inflammation of RAW-264.7 cells and of primary intraperitoneal macrophages (105). One of the mechanisms is the activation of G-protein coupled receptors (GPR), ea. GPR120 inhibition of Toll-like receptor 4 (TLR4)-mediated inflammatory response, which blocks NFκB activation. The other is mediated by nuclear receptors, particularly PPARs-α/γ. DHA binds to PPARs with high affinity resulting in the activation of anti-inflammatory cascades (106), which appears to be responsible for the beneficial health effects (97). The inhibition of NFκB-mediated pro-inflammatory activity (107) is the common mechanism of immunomodulation by n3-PUFAs, being DHA more effective than EPA in reducing LPS-n3-PUFAs induced inflammatory cytokine production by macrophages (108).

n3-PUFAs are incorporated into phospholipid bilayers and in human atherosclerotic plaques. Their incorporation is associated with a reduction in the number of foam- and T cells, and a decrease in inflammation (109). The increased incorporation of n3-PUFAs in membranes affects both the innate and adaptive immune responses, impairing the maturation of dendritic cells and the function of macrophages, as well as the polarization and activation of T and B cells (110–112). It is well known that n3-PUFAs compete with n6-PUFAs for being incorporated into cell membranes and for the active sites of COX-2 and Lipoxygenase, resulting in the production of less potent pro-inflammatory or even anti-inflammatory mediators, such as the 3-series of prostaglandin and thromboxane (113). Resolvins reduce also neutrophil-derived ROS production, favoring neutrophil apoptosis and clearance by macrophages, and inhibit chemokine signaling (114). The partial agonist/antagonist activity of Resolvin E1 (RvE1) on the leukotriene B4 receptor on polymorphonuclear cells (PMNs), inhibits NFκB activation, reduces release of pro-inflammatory cytokines and reduces infiltration by PMN (115). Moreover, RvE1 reduces TNFα and IFNγ presence in the aortic wall, decreases the levels of the inflammatory marker CRP and reduces macrophage infiltration of the intima. Thus, RvE1 attenuates atherosclerosis and atherosclerotic plaque formation (116).

Aging is associated with the activation of inflammatory signaling pathways (117, 118), which can be targeted by specific nutrients with anti-inflammatory effects, such as n3-PUFAs (119, 120). In the brain, the main n3-PUFA is DHA, representing 12–14% of total fatty acids (121). Aging and neurological disorders are associated with decreased levels and turn-over rate of brain n3-PUFAs (122–125). In aged mice, n3-PUFA supplementation and diets enriched in DHA have been reported to revert age-induced spatial memory deficits and impairment on learning and memory (126–128). In older adults, a low consumption of n3-PUFAs and decreased erythrocyte DHA levels are associated with cognitive impairment (129, 130). Dietary supplementation with DHA is positively correlated with an improvement in declarative memory test performance, improved cognitive function (131, 132) and a lower risk of developing neurological disorders (133). The probable mechanisms by which n3-PUFAs mediate their effects in the resolution of age-related neuroinflammation are the increased synthesis of n3-PUFA-derived RvD1 and decreased n6-PUFA-derived oxylipins, displaying an anti-inflammatory profile (134, 135).

To recapitulate, the evidence indicates that n3-PUFAs and their bioactive metabolites have immunomodulatory and anti-inflammatory properties. Potential cardioprotective lipid mediators, through multiple mechanisms, including changes in cell membranes composition, and modification of both cell signaling and gene expression, shift the pattern of lipid metabolites toward a more anti-inflammatory metabolite profile. Dietary habits may be essential regulators of the inflammatory profile and promote healthy aging, reinforcing the recommendation of a n3-PUFA rich diet.

## The Impact of Psychological and Social Stress in the Inflammatory Response

The long term chronic psychological stress is increasing among the world’s population (136). Its circuit arises at high cortical centers through the limbic system to the hypothalamus, where corticotropin-releasing factor (CRF) is produced, which is responsible for inducing the pituitary gland to liberate adrenocorticotropic hormone (ACTH) that signals the adrenal cortex to synthesize and secrete glucocorticoids (GCs) (137). Stress also activates the sympathetic nervous system (SNS), particularly the adrenal medulla, activating chromaffin cells to produce epinephrine (EPI), a main stress hormone along with GCs. The latter plays a key regulation feature inhibiting the hypothalamic-pituitary-adrenal (HPA) axis through negative feedback at the pituitary gland, hypothalamus, and medial prefrontal cortex, reducing CRF secretion [rewieved in (138)].

### Stress and Epigenome Changes

The interplay of social and environmental stressors induces inflammation through multiple biological mechanisms, including epigenetic factors (139). Studies in rats show that the methylation patterns of genes involved in the stress response, such as the glucocorticoid receptor (Nr3c1) and CRF, can be modified by psychosocial factors from early childhood (140). Similarly, early life adversity induces acute and long-lasting epigenetic modifications in Nr3c1 genes, regulating HPA axis and cytokine production, reinforcing the importance of the activation inputs during critical periods of development (137, 141).

### Stress and Immune Response

Acute short-term emotional stress, such as speaking in public, leads to a transient increase in circulating inflammatory biomarkers and natural killer (NK) cells by the SNS catecholaminergic activity (142). On the contrary, chronic stress results in a reduction of cytotoxic NK activity, determining a poorer response to cytokines (143). Therefore, stress appears to have short term beneficial immune effects, whereas chronic stress in the absence of immune challenge has the opposite effect (138, 144), activating constantly the HPA axis with the consequent persistent elevation of systemic GCs and reduction of NK cell responsiveness to cytokines (143), affecting the balance of the T helper cell type 1/type 2 (Th1/Th2) cytokine networks, predisposing to a wide range of diseases (145). The stress magnitude has been associated with IL1β mRNA overexpression in peripheral PBMCs, providing a molecular mechanism by which psychological stress is translated into an immune system response (146, 147).

### Chronic Stress and Chronic Inflammation

When stress becomes chronic, such as in depression, there is a maintained overproduction of inflammatory cytokines, which have been associated with GCs resistance. Immune cells become less sensitive to their anti-inflammatory effects because of their persistent secretion, leading to chronic low-grade inflammation (147, 148). Activation of the innate and adaptive immune system by chronic mild stressors increases inflammatory cytokines gene expression, maturation and trafficking of dendritic cells (DC), increased macrophage number and T cells recruitment and activation. Social stressors can induce an increase in inflammatory responses and a state of GCs resistance at different levels (144, 149).

#### Brain Inflammation

The acute repeated social defeat stress (RSDS) and chronic restraint stress (CRS) models induce an inflammatory response that results in neuroinflammation and depressive behavior (150). Stress activates the HPA axis and the sympatho-adreno-medullar (SAM) axis causing neuroinflammation by circulating cytokines that crossed the blood-brain barrier (BBB) at the circumventricular organs and by cytokine BBB transporters. An inflammatory response that promotes BBB permeability, allowing more inflammatory factors entering the brain, including CRF, metalloproteinase-9, IL6, and TNFα (150). Additionally, microglia produce chemokines that attract monocytes into the brain (150).

#### Endothelium Inflammation

Activation of SNS and HPA axis through continuous psychological stress dysregulate cytokine production, and together with the stress hormones corticosteroids and catecholamines, can affect endothelial adhesion molecules, causing endothelial damage (138). Corticosteroids could facilitate the infiltration of monocytes by increasing the expression of IL1 and IL6 receptors on endothelial cells. These monocytes and lymphocytes, after attaching to such sites, would commence the process of infiltration into the wall vessels, leading to foam cell formation and thrombotic events (138, 151).

#### Pancreas and Liver Inflammation

Chronic unpredictable mild stress (CUMS) decreases body mass and impairs the metabolism of carbohydrates and lipids. A model for CUMS showed an increased liver and pancreas protein-lipid peroxidation and protein oxidation (152). High ROS production in both organs could be a result of a response mechanism to stress at the cellular level. In the liver, protein oxidation can be due to the regulation of metabolic impairments by GCs and EPI (152). The antioxidant system of the liver is in general more efficient than the pancreas. However, it is insufficient to clear the reactive species increased as consequence of chronic stress, which could be due to alterations in the antioxidant enzymatic activity (138).

### Chronic Stress and Aging

Altogether, stress appears to have short term beneficial effects on the immune function, whereas chronic stress (138, 144) activates persistently the HPA, elevating systemic GCs, and impairing the cytokine balance. The overproduction of inflammatory cytokines lead to GCs resistance driven by immune cells that lose their sensitivity to GCs, leading to a state of chronic low-grade inflammation (138, 145). This GCs imbalance, shares common features with aging, mediating an enhanced neuroinflammatory priming (153). The presence of psychological stress potentiates the defective immune response observed in aging, which at the same time conditionate an exaggerated sickness response to immune challenges (such as chronic stress). Thus, chronic stress contributes to the phenomenon of inflammaging, which promotes the development of several age-related pathologies, including atherosclerosis and diabetes among others [reviewed in (154)]. Additionally, there is an impairment of the antioxidant defense system to manage ROS production after chronic stress, resulting in the damage of various tissues (138). In addition, people exposed to chronic stress age rapidly, showing a faster telomere shortening in their cells (155–157). On the other hand, epigenetic changes acquired during critical developmental stages could shape chronic stress-response along the lifespan, either promoting or reducing pathological aging (139, 140).

## Inflammatory Response Induced by Drug Abuse

Substance abuse, such as alcohol and drugs, are important triggers of chronic inflammatory processes (158, 159). The effects of alcohol on human health are complex and depend on multiple factors. However, many of those factors are associated with the generation of immunosuppression and increased morbimortality in heavy users. Those effects, which have been previously reviewed by Goral et al. (160) will not be discussed in this review. Here, we will describe the effect of cocaine and methamphetamine abuse. Both drugs are potent psychostimulants that, when repeatedly consumed, significantly disrupt the functioning of the CNS, and modify the regulation of the immune response, leading to a chronic neuroinflammatory state (161). In general, it is known that drug abuse, among other factors, increases NFκB transcription of multiple proinflammatory genes that spread across brain cell types further amplifying of NFκB transcription, as has been reviewed by Crews et al. (162).

### Cocaine

Cocaine (benzoylmethylecgonine according to the International Common Denomination) is a strong stimulant tropane alkaloid that acts by modulating the catecholaminergic neurotransmitter dopamine. Studies of the striatum of mice after the administration of various drugs showed that 1 h after administration of 25 mg/kg cocaine, there is a significant increase in gene arrays for Hypoxia-inducible factor 1 (HIF-1), transcription factors, and cytokine receptors (IL6r, TNFα). Two hours after cocaine administration, there is an increased gene expression for various TNF receptors, inducible NO synthase (iNOS) and adhesion molecules (163). In the nucleus accumbens of mice stimulated with cocaine, there is a significant increase in matrix metalloproteinase 28 (MMP28), Macrophage Colony Stimulating Factor (MCSF) and Major Histocompatibility Complex II (MHC-II) (164). The brain of human subjects consuming cocaine shows an increased density of macrophages and activated microglia (165). Cocaine induces the activation of microglia through the endoplasmic reticulum stress and autophagy pathways (166). Studies of human and rodent immune cell populations after cocaine administration show decreased numbers of T lymphocytes, modulation of NK activity and cytokine production (167).

Among brain glial cells, astrocytes are the most abundant, and perform critical functions, being involved in neurogenesis, promotion of neuronal survival, elimination of free radicals, and the production of NO to maintain neuronal homeostasis (166). Nevertheless, astrocytes can also be activated by toxic stimuli, leading to a new phenotype called “reactive astrocytes”, similar with the changes observed after inflammatory activation. This phenomenon has been described in various neuropsychiatric disorders, such as Alzheimer’s and Parkinson’s disease, amyotrophic lateral sclerosis and multiple sclerosis (166). The reactivity of astrocytes to toxic stimuli, such as cocaine, infection or disease, potentiates the neuroinflammatory process (168).

### Methamphetamine

Methamphetamine (desoxyephedrine; METH) is a synthetic adrenergic agonist with psychostimulatory effects, structurally related to the ephedrine alkaloid and adrenaline. Studies on the effect of METH are limited. However, it has been determined that its abuse affects the immune response. Animals exposed to both acute and chronic METH use show alkalization of normally acidic organelles in immune cells, inhibition of antigen presentation, and impairment of phagocytosis (169). METH also generates mitochondrial oxidative damage, dysfunction of T lymphocytes and decreased production of antibodies and cytokines (159).

METH has effects in various tissues (170). In the lungs, the number of T lymphocytes decreases compared with that of untreated animals, indicating a reduction in circulating CD3+ cells, and levels of IL6 and IL10 increases. In the spleen, recruitment of PMN and the number of Ly-6G+ and F4/80+ are increased, whereas CD3+ cells are significantly reduced. In addition, levels of TNFα, IFNγ, IL6, and IL12 are higher than those of control mice. In the liver, there is an increase of T lymphocytes and macrophages, hepatocellular atrophy, and increased levels of IFNγ, TNFα, IL1β, -4, -6, -10, and -12 in the group exposed to METH compared with control animals (170).

In the CNS, METH can induce the activation of calpains and caspases; the production of ROS with the subsequent induction of oxidative stress, and the release of high amounts of glutamate, causing excitotoxicity (171). Recently, Raineri et al. reported that METH induces activation of astrocytes and microglia, increasing the levels of IL6 and TNFα mRNA and its receptor (TNFR1) in the mouse striatum and hippocampus (172, 173).

### Drug Use and Aging

Medical advances have resulted in the increment of the average life expectancy in developed countries. The aging of the population is associated with an increase in the number of older people using drugs of abuse. From 2000 to 2012, the number of cocaine users aged 55 or older that required treatment for drug addiction in the US increased by 63% (174, 175). Aging is associated with low-grade basal inflammation that can be compounded by substance use. As cocaine exposure is associated with elevated inflammation and altered immune functioning, the presence of cocaine use disorder might exacerbate inflammatory processes in aging adults (176). A recent report by Soder et al, compared the levels of inflammation (through the neutrophil to lymphocyte ratio) in older adults with cocaine use disorder (CUD) and in healthy older adults, finding that the group with CUD had a significantly higher baseline level of inflammation (176). The use of illegal drugs such as cocaine or methamphetamine has not been shown to affect cognitive function in older adults at the clinical level. However, the evaluation of the cognitive function of young drug users reveals a decreased performance compared with healthy young people. In fact, the cognitive function of young drug users is similar to that of adults older than 60 years of age (174, 177, 178).

In summary, both cocaine and METH can directly impair the immune response, induce the activation of glial cells and stimulate the release of pro-inflammatory mediators in the CNS. All those effects cause relevant changes in glial cell regulation and inflammatory activation, triggering chronic neuroinflammation and potentiating pathological aging.

## Induction of an Uncontrolled Inflammatory Process by Air Pollution

Air pollution has become an important threat to public health. Air pollutants consider a mixture of gases such as nitrogen oxides (NOx), sulphur oxides (SOx), tropospheric ozone (O3), volatile organic compounds (VOCs), and particulate matter (PM) (179). PM can enter the respiratory tract leading to severe *in situ* damage as well as inducing additional systemic effects (180). The World Health Organization (WHO) suggests a maximum annual exposure of 10 µg/m³ of PM2.5, however, the exposure of 90% of the world’s population exceeds the proposed limit (181). Exposure to air pollutants is associated with increased morbimortality associated with respiratory, cardiovascular, metabolic, neurological, carcinogenic and autoimmune diseases (17, 182–184). Inflammation is the main pathophysiological mechanism induced by air pollutants.

### Oxidative Stress

In terms of the molecular and cellular mechanism induced by pollutants, PM and SOx can generate ROS, inducing oxidative stress, together with mitochondrial dysfunction and the consequent energy deprivation (185–187). As a direct consequence, NFκB and MAPK inflammatory pathways are activated, triggering an innate immune activation (188, 189). Despite the attempts to resolve the inflammatory event, the outcome appears to be an imbalance in lymphocyte homeostasis and immune system dysregulation, with inhibition of Th1 and Treg lymphocytes (190). There is also an increase of Th2 lymphocytes and recruitment of eosinophils, resulting in respiratory disorders such as asthma (186, 191, 192). In parallel, PM deactivates the nuclear factor erythroid 2 pathway (Nrf2), involved in antioxidant regulation and prevention of oxidative stress, a necessary process for the resolution of inflammation. Therefore, to maintain oxidation-reduction reactions becomes impossible, becoming a breaking point towards increased ROS production and the non-resolution of the inflammatory event (193).

### Activation of the Aryl Hydrocarbon Receptor

Another mechanism of action of pollutants is the activation of the aryl hydrocarbon receptor (AhR) by toxic agents. The binding of PM to AhR increases circulating Th17 and decreases Treg lymphocytes. Increase in Th17 associates to the release of IL17, promoting an abrupt increase of Th2 lymphocyte response. These changes promote the dysregulation of the immune response associated with the development of autoimmune processes (193). Aberrant increases in Th17 may result in increased inflammation, with consequences such as asthma and acute respiratory failure syndrome (ARDS), due to neutrophil infiltration and tissue damage (194). Studies suggest the existence of a decline in Treg levels and, therefore, an inability to suppress Th1, Th2 and phagocyte responses (195, 196). In addition, exposure to PM has been associated with fibrotic events, where IL17 increases synthesis and secretion of collagen in the lung parenchyma (197, 198). In addition, it has been described that PM also induces the expression of TGFβ, directly promoting fibroblast differentiation, which could also induce collagen deposition followed by a lower antifibrotic process in the liver (199).

### Epigenetic Regulation

Pollutants may promote direct DNA damage through oxidation of nitrogenous bases. Hu and Yu described in a 2019 paper different mechanisms and changes in miRNA expression that comprise specific targets of DNA methyltransferases, which can impair the methylation of tumor suppressor genes (200). Furthermore, urban populations show increased levels of mitochondrial methylation genes due to PM exposure (201). There is evidence of the existence of methylation, acetylation and phosphorylation of histones H3 and H4, markers found in genes involved in the activation of immune cells and cardiovascular diseases (200, 202–205). Altogether, air pollutants can generate DNA adducts promoting carcinogenesis and deteriorate telomerase activity, as reviewed by Martens and Nawrot (2016), and contributing to continuous DNA damage and premature aging (206, 207).

### Temporal and Concentration Effects Over Inflammatory Mediators

In vivo studies suggest that the inflammatory activation is dose- and time-dependent. Mice exposed to PM show that both variables are determinant for the outcome. However, inflammatory effects and major genetic changes appear to be especially dependent on the exposure to high concentrations of PM. One possible explanation is that a prolonged exposure could induce an adaptive response of the inflammatory activation (208), which may be mediated by the inactivation of the Nrf2 pathway, generating a loss of antioxidant capacity and deregulation of the immune system (193). The resolution appears to depend on the exposure context. Acute exposure would result in high levels of ROS and damage, whereas prolonged stimulation, even a low-grade one, generates a constant production of ROS and chronic low-grade inflammation (187), consequent with the potentiation of disease risk and an epigenetic age acceleration (206), promoting pathological aging.

Direct causes of the deregulation of the inflammatory resolution process resulting from inhaled contaminants are still unknown, however, the burden of associated chronic diseases is expected to increase. It is mandatory to intensify environmental policies specifically in lower-middle-income countries in prevention of the development of inflammatory conditions and the subsequent chronic diseases.

## Aging, Epigenetic and Immuno-Inflammatory Imbalance

Aging, characterized by a progressive loss of cellular functions, is an inevitable physiologic process inherent to all living beings (209). The number of older adults is increasing. During the next 30 years, up to 22% of the world population will be older than 60 years (210). This demographic change is accompanied by a higher incidence of NCDs accumulated in the aging population (211). Therefore, various strategies have been proposed to improve the health and quality of life of older adults (212), along with recommendations for the development of Public Policies that support the fiscal expenditure resulting from NCDs (213).

One of the most studied events of aging is the impairment of the immune system, characterized by an aberrant-increased activation of the innate immunity (214, 215), and high levels of circulatory inflammatory mediators that establish an inflammatory environment, and a decrease of the adaptive immune response (216, 217) and a decrease of the adaptive immune response (214) due to this low-grade chronic inflammation (214, 218), which together would promote the inflammaging phenomenon (219). Interestingly, it is proposed that age would not be the cause per se of these diseases associated with aging (214). Thus, there is a deterioration of the immune system’s response to external stimuli, which depends on the individual’s history (218). Also, several epigenetic mechanisms can modulate the immune response in aging, enhancing changes in intercellular communication that could perpetuate inflammatory events (220). On the other hand, it is described that epigenetic clocks would be useful to analyze mechanisms associated with this environmental influence (221). Finally, they would be capable of modulating the immune response in aging, enhancing changes in intercellular communication that could perpetuate inflammatory events (220).

### Aging and Systemic Inflammation

Multiple age-dependent changes play important roles in the promotion of NCDs, with increased oxidative stress standing out as one of the main mechanisms. Over the last two decades, evidence has revealed that increased oxidative stress and inflammation are involved in various NCDs such as Alzheimer‘s disease (219), rheumatoid arthritis (222), cardiovascular diseases (223, 224), and cancer (225), among others. Also, recent studies propose that the activation of NFκB signaling pathways could be the main driver of these associations (226–229). Interestingly, De Almeida et al. showed different sources of low-grade chronic inflammation that promote cardiovascular disease (226). In the CNS, high levels of ROS lead to the activation of astrocytes and microglia, further increasing the overproduction of ROS and proinflammatory cytokines that promote the development of neurodegenerative changes (217, 230, 231). In fact, several systemic biomarkers appear to be associated with neuroinflammation and the development of CNS diseases associated with aging (230). These modifications trigger the phenotype of senescent or aged cells characterized as SASP (216, 232) extensively studied in the context of the deleterious effects of aging. However, SASP is also essential for remodeling and promoting wound healing, which requires a strict control of the inflammatory response, thus avoiding the induction of cell aging phenotypes that contribute to the development of chronic inflammatory diseases (233).

### Mechanisms Associated With the Immune Imbalance

The immune imbalance in aging occurs due to various alterations in cellular behavior and phenotype, which cause functional deficiencies in immune cells (3). For example, this context induces polarization of macrophages towards an inflammatory phenotype characterized by strong activation of the inflammasome (234). Thus, these events could induce IL1β and TNFα release, changes in the chemoattraction of neutrophils mediated by the reduction of the intercellular adhesion molecule 1 (ICAM-1) expression, and the aberrant activation of the phosphoinositide lipid kinase-3 (PI3K) (235). Also, there is a decrease in the expression of pattern recognition receptors (PRR), which leads to the activation of proinflammatory signaling promoting tissue damage (215, 216). Finally, the reduced level of certain hormones due to the impaired hypothalamic function causes the loss of muscle mass and an increase in adipose tissue, further contributing to the release of inflammatory cytokines and changes in metabolism (236). Despite the remarkable effort being made to understand the basis of the processes underlying the inflammatory imbalance during aging, it is not fully understood.

### Role of Epigenetics in the Immune Imbalance

In aging, there are cumulative epigenetic changes that promote low-grade inflammation (220, 237), including a decrease in the global genome methylation, with increased methylation in specific regions, as those with repressive histone marks of CD8+ and CD4+ T cells (238) and bivalent chromatin domains (239) and histone acetylation and methylation. However, the influence of genomic methylation during aging remains undetermined (237). Several studies correlate the methylation of multiple sites on CpG islands with the increase of the low-grade inflammation marker, CRP (220, 232, 240). Nonetheless, Stevenson et al. propose that the DNA methylation could be better associated with the low-grade chronic inflammation than CRP (237). In addition, the age-related mitochondrial dysfunction, with the resulting oxidative stress and decreased ATP production (241), affect the expression and activity of DNA methyltransferases, which are responsible for maintaining the methylation pattern of DNA (242). The reduced methylation results in the demethylation of the TNFα promoter in leukocytes and macrophages (243) and the adhesion of immune cells to the endothelium (244). Also, many epigenetic events contribute to the differentiation of proinflammatory T cells, Th17 (220), which can compromise immunocompetence, associated with repression of differentiation of immune cells, loss of Treg function (240) and the alteration of the hematopoietic stem cells differentiation (245).

Thus, epigenetic mechanisms appear to have a major role in the inflammatory imbalance, which are associated with the accumulation of damage in time that ultimately leads to the perpetuation of a constant inflammatory response.

## Modulation of the Inflammatory Activation Through Physical Exercise

According to the WHO, 60% of the world population is sedentary, lacking the benefits of physical exercise (246). Conditions such as sedentarism, unhealthy diet, overweight, obesity and aging induce chronic low-grade inflammation. Physical exercise increases the anti-inflammatory potential and reduces the pro-inflammatory effect (247). This equilibrium is partly modulated through TLRs (248), which are fundamental for the recognition of PRRs, including the damage-associated molecular patterns (DAMPs) and the induction of an inflammatory response in the absence of pathogens.

### Anti-Inflammatory Exercise

There is evidence that in young people, physical exercise decreases TLRs expression, co-stimulatory molecules CD80/CD86, and MHCII (248, 249) in CD14+ monocytes. Physical exercise also affects the adipose tissue. Exercising reduces TLR4 mRNA expression and TNFα production in adipocytes (250, 251) in obese mice. Chronic physical exercise decreases TNFα and TLR4 gene expression in the skeletal muscle (252). The evidence suggests that obesity- or cerebral ischemia-induced neuroinflammation, which are associated with the overexpression of TLR2 and TLR4, may be reduced by physical exercise through the reduction of TLRs expression as well as their downstream signaling molecules (TNFα, IL1β, MyD88, TRAF6, 552 TAK1, and NFκB), together with the reduced microglial activation (253, 254). There is evidence that cigarette smoking induces inflammatory status [reviewed in (255)]. However, exercise training reduces smoke-induced inflammation. In that sense, training for 30 min with endurance exercise for 5 days in smoke-exposed mice demonstrated that therapeutic exercise training significantly reduces the expression of IL1β and TNFα mRNA in rectus femoris (256).

Physical exercise has been used as a therapeutic tool in chronic pathological conditions. In that sense, obese older adults (body mass index 38 ± 2 kg/m2; 69 ± 1 years) undergoing an exercise program consisting in physical therapy, endurance, and resistance for 90 min, 3 days per week, show a reduced expression of TLR4, IL6, and TNFα mRNA in skeletal muscle (257). In older adults, 8-week physical exercise reduces the expression of TLR4 and TLR2, as well as TLRs downstream mediators, such as MyD88, p65, pp38, TRIF, IKKi/IKKϵ, IRF3, and pIRF7 in PBMCs (258). Similarly, dendritic cells from multiple sclerosis patients undergoing an exercise (endurance and resistance) program for 12 weeks reduce TNF*α* and MMP9 secretion when stimulated with a TLR4 ligand (LPS in combination with IFN*γ*, or a TLR7 ligand) (259), suggesting that long-term physical exercise decrease TLR responsiveness.

### Pro-Inflammatory Exercise

On the other hand, high-intensity physical exercise in untrained individuals induces inflammation, resulting in the increased expression of TLR4, AP1, NFκB, and p65 in mice myocardium and in adipose tissue (260–262). Physical exercise associated with eccentric contractions causes expression of TLR and NFκB in skeletal muscle and liver in rats (263, 264). Furthermore, this phenomenon induces muscle damage, which can increase chemotaxis, attracting NK, CD8+ 559 T cells, macrophages and neutrophils to the site of injury, promoting the production of COX560 2, iNOS, monocyte chemotactic protein-1 (MCP-1), TNFα, IL6, and IL1β, in addition to the production of ROS and the activation of NFκB (265, 266). In healthy young males, one session of intense endurance exercise (1 h intense cycling immediately followed by 1 h intense running), increases plasmatic concentrations of IL6 and IL10, in addition to increased gene expression of proinflammatory IL1 receptor (IL1R) and TLR signaling pathways. Moreover, plasma myoglobin changes in correlation with neutrophil TLR4 gene expression (r= 0.74), suggesting that their transcriptional activity was particularly induced by DAMPs (267). Therefore, inflammation and muscle damage are mainly associated with the type and intensity of the exercise, with loads that exceed individual physical abilities.

### Exercise, Epigenetic Regulation, and Inflammation

Chronic physical exercise generates epigenetic modifications. The physical exercise associated with an energy expenditure >500 kilocalories per week, results in hypomethylation of the IL10 gene and hypermethylation of the TNFα gene (268), with an inverse correlation between TNFα methylation and TNFα mRNA expression (269). The methylation of the caspase recruitment domain (ASC) of the apoptosis-associated speck-like protein gene, the main regulator of inflammasome and promoter of the activation of IL1β and IL18, decreases with aging. However, older adults who maintain physical exercises regularly express higher levels of ASC methylation than subjects not exercising, which would imply a decreased release of inflammatory cytokines (270–273). Similarly, in review a 6-month walk training can induce hypermethylation of the NFκB-2 gene, suppressing inflammation through the inhibition of the NFκB pathway (274).

## Discussion

As life expectancy increases, age-related diseases thrive. Aging is a complex multifactorial process of molecular and cellular decline that renders individuals susceptible to disease and death. Maintenance of cell integrity, cell metabolism and host-defense mechanisms are tightly regulated by the surrounding microenvironment. A growing body of evidence in different biological models has contributed towards identifying biological mechanisms that ward off structural and functional deterioration. These data offer us insights into healthy aging. Molecular integrity of the genome, telomere length, epigenetic stability, and protein homeostasis are all features linked to more youthful stages (regardless of the age), associated with mitochondrial fitness, metabolic regulation, efficient intercellular communication, stem cell renewal, and regenerative capacity in tissues. A good understanding of the environmental and endogenous mechanisms that underlie age-related normal and deleterious changes, and how these pathways interconnect, remains a major challenge for slowing pathological aging while extending older adults’ healthy lifespan.

**Figure 1:**
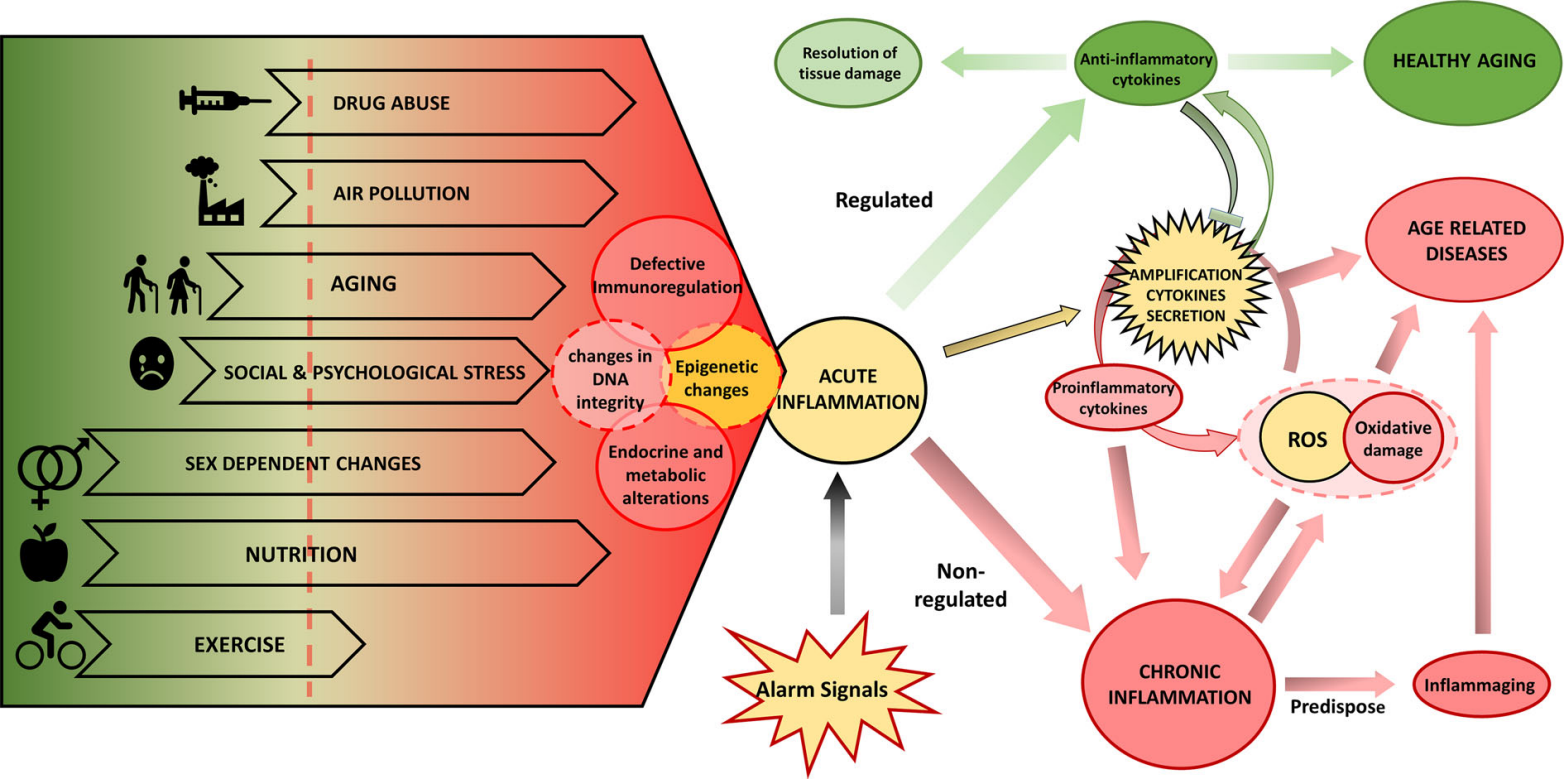
Biological and environmental factors determining the inflammatory response and the aging phenotype. Endogenous and environmental factors can be mostly beneficial (in green) and deleterious (in red) or can have both beneficial and deleterious effects depending on the specific context. The interplay of lifespan endogenous and environmental factors regulates the aging phenotype depending on DNA damage, epigenetic changes, and inflammation. These drivers can induce functional aging hallmarks: changes in endocrine and metabolic regulation, and defective immune regulation that will further determine the response of the individual. In yellow we show processes that can participate in both protection and damage. Exposure to various alarm signals induce an acute inflammation that, when associated with deleterious environmental and biological factors, potentiates chronic inflammation, which can be further promoted by excess ROS production and oxidative stress that results from mitochondrial dysfunction or NOX2 activity, leading to inflammaging and eventually to age-related disease. On the contrary, in the presence of protective environmental and biological factors, the initial inflammatory activation will be resolved and lead to a healthy aging process. ROS, reactive oxygen species.

The study of the environmental influence on the development of complex-chronic diseases shows that in addition to genetic predisposition, the pathogenesis is promoted by changes in metabolism and behavior, cellular environment, and epigenetic regulation patterns. The type of nutrient, or environmental cytokine milieu dramatically affects not only the homoeostasis of tissues but also of complete organs and even of the whole individual. Thus, tissue stress, malfunction, and damage may induce inflammation alarm responses, which result either in resolution of tissue damage, restoration of normal cell function or development of chronic disease (**Figure 1**). Older adults often present inflammaging, characterized by increased levels of pro-inflammatory cytokines IL1, IL6, IL8, TNFα/CRP (275). However, the cellular sources of these cytokines are partially unknown. The increased inflammatory cytokines have been proposed to be a driver of unsuccessful aging (increased morbidity, degenerative processes, or frailty) and shortened health-span. The inflammatory scenario is complex and occurs in response to various internal and environmental stimuli (**Figure 1**) mediated mainly by a high level of pro-inflammatory cytokines. Indeed, in healthy aging, increased production of the anti-inflammatory cytokines TGFβ and IL10, can regulate the pro-inflammatory state (276, 277).

Research into the impact of environmental factors on inflammaging is at an early stage and the involved mechanisms are not completely understood. Several hypotheses have been developed to explain the chronic inflammation: aging-related increase of stress (278) and oxidative stress (279), DNA damage in senescent cells [reviewed in (280)], and stem cell aging (281). The proposed mechanisms are likely interdependent, resulting in the generation of ROS causing oxidative damage and amplification of the cytokines secretion, thus perpetuating a vicious circle of systemic inflammation where tissue injury and healing mechanisms proceed in parallel while damage slowly accumulates over the lifespan of the individuals. Endocrine and metabolic alterations are linked to the shift towards a pro-inflammatory profile, which could explain some age-related pathologies, such as Alzheimer’s and Parkinson’s disease, osteoporosis, diabetes, cancer, and frailty (282, 283).

Regarding stress-induced immune modifications, new evidence suggests that cross talk signals between the CNS, endocrine and immune system are required for optimal response to stress [discussed in (284)]. Various stressors can affect the activity and regulation of immune cells *via* direct regulation by the autonomic and peptidergic system or through the release of neuroendocrine mediators. Moreover, neuronal catecholamines modulate immune cell functions. These interactions are bidirectional, cytokines produced by immune cells, such as IL1, can modulate the production of corticotropin-releasing hormone (CRH) by the hypothalamus. Chronic diseases are favored by some modern living conditions, such as the intake of high-caloric foods and the low level of physical activity, or endogenous signals produced by the chronic stress of modern life. There are many challenges in conducting research on biosocial processes, which will define novel disease-trigger factors.

Tailor-made approaches will depend on genetics, epigenetics and a constellation of factors depending on the historical as well as the present exposure to the environment. Although environmental factors also express themselves as epigenetic changes, the combinatorial effect of the multiple factors generates complex patterns of epigenetic regulation, and the concomitant exposure to environmental factors can further modify the individual response.

## Author Contributions

All authors contributed equally on the conception of the work, the analysis of literature and preparing the content of the review. RBe drafted and organized the manuscript. All authors contributed to the article and approved the submitted version.

## Funding

This work was partially supported by Project FONDECYT 1171645 from CONICYT Program of Chile and the Santander Universia Research Award in aging research (RBe). SB, YJ, and FG acknowledge partial support from CONICYT-PFCHA/Doctorado Nacional 2019-Folio 21191070, 21191025 and 21190421, respectively. RBa and JM acknowledge partial support from CONICYT-PFCHA/Doctorado Nacional 2020-Folio 21201751 and 21201210, respectively.

## Conflict of Interest

The authors declare that the research was conducted in the absence of any commercial or financial relationships that could be construed as a potential conflict of interest.

## Abbreviations

ACTH, adrenocorticotropic hormone; Ahr, aryl hydrocarbon receptor; AMPK, 5’ AMP-activated protein kinase; ARDS, acute respiratory distress syndrome; ASC, caspase recruitment domain; BBB, blood-brain barrier; cAMP, cyclic adenosine monophosphate; CNS, central nervous system; COX-2: cyclooxygenase-2; CREB, cAMP response element-binding; CRF, corticotropin releasing factor; CRS, chronic restraint stress; CUMS, chronic unpredictable mild stress model; DAMPs, damage-associated molecular patterns; DC, dendritic cells; DHA, docosahexaenoic acid; EPA, eicosapentaenoic acid; ERα/β, α- and β-estrogen receptors; GCs, glucocorticoids; GPR, G-protein coupled receptors; HPA, hypothalamic-hypophysis-adrenal; hsCRP, high sensitivity C-reactive protein; ICAM-1, intercellular adhesion molecule;,IFN, interferon; IL1Rα, IL1 receptor α; IL1β, interleukin 1β; iNOS, inducible NO synthase; LGCI, low-grade chronic inflammation; LPS, lipopolysaccharide; MAPK, mitogen activated protein kinase; MCP1, monocyte chemotactic protein-1; MCSF, macrophage Colony Stimulating Factor; METH, methamphetamine; MHC-II, major histocompatibility complex II; MMP28: matrix metalloproteinase 28; NCDs, noncommunicable diseases; NFκB, nuclear factor kappa-light-chain-enhancer of activated B cells; NK, natural killer; NO, nitric oxide; NOx, nitrogen oxides; Nrf2, nuclear factor erythroid 2 pathway; O3, tropospheric ozone; PBMCs, peripheral blood mononuclear cells; PI3K, phosphoinositide lipid kinase-3K; PM, particulate matter; PMNs, polymorphonuclear cells; PPAR, peroxisome proliferator-activated receptor; PRR, pattern recognition receptors; PUFAs, polyunsaturated fatty acids; ROS: radical oxygen species; RSDS, repeated social defeat stress; RvE1, resolvin E1; SAM, sympatho-adreno-medullar axis; SASP: senescence-associated secretory phenotype; SDR, social disruption; SNS, sympathetic nervous system; SOx, sulphur oxides; TGFβ, transforming growth factor β; TLR4, toll-like receptor 4; TNFR1, TNFα receptor 1; TNFα, tumor necrosis factor-α; Treg, regulatory T cells; VOCs, volatile organic compounds; WHO, World Health Organization.
